# Poly 3-Hydroxybutyrate 4-hydroxybutyrate (P34HB) as a Potential Polymer for Drug-Eluting Coatings on Metal Coronary Stents

**DOI:** 10.3390/polym14050994

**Published:** 2022-02-28

**Authors:** Yihui Jian, Yufang Zhu

**Affiliations:** 1 School of Materials and Chemistry, University of Shanghai for Science and Technology, Shanghai 200093, China; 2 AccuPath Medical (Jiaxing) Co., Ltd., Jiaxing 314006, China; 3 Shanghai Institute of Ceramics, Chinese Academy of Sciences, Shanghai 200050, China; zjf2412@163.com

**Keywords:** poly 3-hydroxybutyrate 4-hydroxybutyrate, P34HB, drug-eluting coatings, drug-eluting stents, coronary stents

## Abstract

Drug-eluting stents (DES) are a main interventional therapeutic instrument to treat coronary diseases. Degradable polymers such as polylactic acid (PLA) for coatings that only degrade into small molecules in human bodies have been developed for coating polymers, but most coatings often lack ductility and can be easily peeled off from the stents after balloon expansion. In this study, biodegradable poly 3-hydroxybutyrate 4-hydroxybutyrate (P34HB) with good ductility was proposed to be a latent polymer for drug-eluting coatings on the stents. Using P34HB-1 (4HB% = 1%wt, Mw: 225,000) and P34HB-10 (4HB% = 10%wt, Mw: 182,000) as two candidates, both P34HB-1 and P34HB-10 exhibited excellent solubility in CHCl_3_. Their drug solutions remained highly stable and did not become turbid over a period of 48 h, and were conducive to batch preparation of uniform drug coatings. Drug coatings made by both P34HB-1 and P34HB-10 on the stents were almost complete before and after dilation by balloon owing to their excellent adhesion and extrusion resistance properties. Furthermore, both P34HB-1 and P34HB-10 had excellent biocompatibility in cytotoxicity and hemolysis tests. However, P34HB-1 drug coatings showed better drug release control than P34HB-10 drug coatings and Firebird2^®^, indicating that P34HB-1 is more suitable for a latent coating polymer of coronary stents.

## 1. Introduction

Coronary disease is common in humans, especially in older persons. There are at least two million people requiring heart disease treatment in America every year, and about one million people needing treatment each year in China.

PCI (Percutaneous Coronary Intervention) is a main coronary disease treatment method because of its advantages of micro-trauma and good therapeutic effect. Since restenosis has been a great challenge for interventional therapy, coronary stenting has significantly reduced the restenosis rate from 30~60% in the era of balloon angioplasty to 10~30% and has become the most commonly used PCI technique. At the beginning of the 21st century, drug-eluting stents such as Cypher^®^ and Taxus^®^ reached a new milestone. Drug-eluting coatings are deposited onto bare stents so that drug can be released slowly, and the incidence rate of restenosis can be decreased greatly, but the inflammatory reaction of non-degradable polymers after stent implantation can lead to insufficient endothelialization and late thrombosis. Biodegradable polymers only degrade into small molecules by hydrolysis or enzymatic hydrolysis mechanism, which do not accumulate and cause cumulative toxicity in human bodies, thereby enhancing their safety [1].

Coatings with biodegradable polymers are becoming more and more prevalent. However, PLA has poor ductility, tends to rupture and can be peeled from the bare stent after the balloon’s expansion [2].

To develop other latent biodegradable polymers is becoming necessary. Polyhydroxy alkanoates (PHAs), with over 150 different monomeric units as constituents, are a family of biopolyesters synthesized by many bacteria [3,4]. Since most natural PHAs are biodegradable, they have been studied extensively for various potential medical applications [3,5]. Amongst all types of PHAs, poly(3-hydroxybutyrate) (PHB) is used in various fields as it is a biodegradable, biocompatible, and ecologically safe thermoplastic. The unique physicochemical characteristics of these PHAs have made them applicable in nanomedicine, tissue engineering, and other biomedical applications [6]. Most studies have been focused on PHB, the first member of the PHA family to be discovered [7]. PHB was found to have potential applications as an implant material owing to its biocompatibility, yet its brittle mechanical property limited its applications [8]. Poly(3-hydroxybutyrate-co-3-hydroxyhexanoate) (PHBHHx), a new member of the PHA family, has substantially different behavior and is more ductile than PHB due to long chain branching that reduces crystallinity and melting temperature [9]. Poly 3-hydroxybutyrate 4-hydroxybutyrate (P34HB), the newest member of the PHA family, introduces more flexible 4-hydroxybutyrate segments into the 3-hydroxybutyrate segments, and the ductility of the copolymer is improved [10]. Hence, both PHBHHx and P34HB of PHA series may have good adhesion to the stent surfaces after balloon expansion, and the coatings may not fission and peel off.

To date, a few studies have reported the utilization of PHA series for coronary stent drug coatings [11], and it has been shown that the second-generation poly(3-hydroxybutyrate-co-3-hydroxyvalerate) (PHBV) material of PHA series was a potential tissue engineering material and drug release material [12]. For example, Gwenaelle et al. [11] found that using PHBV as a stent coating material, drug release rate was faster. Therefore, a new block copolymer P(HBV-b-LA) was developed, and a dual-layer coating was achieved to optimize drug release rate by constructing PHBV as the bottom layer and P(HBV-b-LA) as the drug loading layer. Such dual-layer coating had good flexibility and stent adhesion, and improved drug stent coating. Peng et al. [13]. prepared hydrophilic insulin–phospholipid complex-loaded PHBHHx nanoparticles for controlled release to reduce insulin administration frequency. They showed a higher bioavailability compared to an insulin solution. Thus, it can be inferred that the third-generation PHBHHx and the fourth-generation P34HB biodegradable polymers of the PHA series could also be used as stent coatings materials. However, our previous studies indicated that PHBHHx had side chain -C_3_H_7_ compared to P34HB, which made PHBHHx much softer than P34HB and easy to melt under hot squeezing conditions (temperature: 60 °C), while P34HB did not melt. In the process of assembling a drug stent with a balloon catheter, a squeezing machine is used to squeeze the stent so that the stent is closely attached to the balloon. To achieve a good attachment effect, a squeezing machine with a high temperature (such as 60 °C or even higher) is used to squeeze the stent. PHBHHx was easy to melt, while P34HB did not. Therefore, in this respect, P34HB is a more suitable candidate for drug eluting stents.

In this study, the fourth-generation P34HB polymers (P34HB-1, 4HB% = 1 wt%, Mw: 225,000 and P34HB-10, 4HB% = 10 wt%, Mw: 182,000) were utilized as coating polymers for drug eluting stents. The solubility and stability of polymer spraying solutions, coating morphologies, drug release performance and biocompatibility of coating polymers were systemically investigated.

## 2. Materials and Methods

### 2.1. Materials

The bare stainless-steel stents used in this study were manufactured by laser cutting on fine tubes, followed by electrochemical polishing. PHBHHx polymer (HHx% = 10%wt, Mw: 200,000) and P34HB polymers (P34HB-1: 4HB% = 1%wt, Mw: 225,000; P34HB-10: 4HB% = 10 wt%, Mw: 182,000) were purchased from Bluepha Co., Ltd. (Beijing, China). Sirolimus (RAPA, high-performance liquid chromatography, HPLC) with purity greater than 98%wt was purchased from Shanghai Jiahe Biological Technology Co., Ltd. (Shanghai, China). All reagents purchased from Sinopharm Group: CHCl_3_ and N-propanol (NPA) were HPLC grade, and another reagent (DMF) was analytical grade.

### 2.2. Stability of Polymer Solutions

P34HB/RAPA (weight ratio of 10/1) was dissolved into the same volume of CHCl_3_, mixed solvents of CHCl_3_/NPA and DMF to form 18 mg/mL solutions, respectively. After being stirred for 4 h, the solubility of the components was observed by eyes. Finally, the solutions were placed statically for 48 h to record stability by eyes.

### 2.3. Coating Procedure

**Figure 1:**
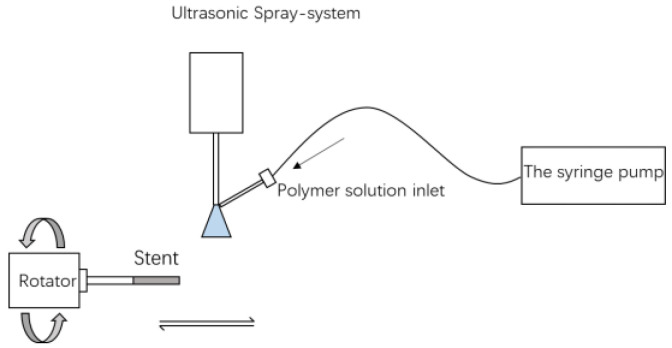
A schematic diagram for the preparation of the polymer coatings on stents.

The ultrasonic spray-coating method of Sono-tek Corporation was used to prepare the polymer coatings on stents as in Figure 1. Before being sprayed, the P34HB solutions containing RAPA were prepared (18 mg/mL of P34HB solutions and 1/10 of drug/polymer ratio) and injected into the syringe pump. The spraying parameters were as follows: 0.08 mL/min of solution flow rate, 0.6 w of nozzle power, 0.5 mL/min of focusing pressure, 15 cycles of mandrel moving times and 0.8 cm/s of mandrel moving speed. After the ultrasonic spray coating procedure, the coated stents were taken out and dried at room temperature.

### 2.4. Observation on Coating Surface Morphologies

The surface morphologies of the drug-eluting stents before and after being squeezed by the machine, and before and after dilation by balloon, were observed on a scanning electron microscope (Model S-3400N, Hitachi Limited, Tokyo, Japan) in a vacuum.

### 2.5. Drug Release Profiles Measurement

For evaluation of in vitro drug release, 15 mL PBS/ethanol (9/1, pH = 7.4) solution was prepared and then placed into a thermostatic oscillator at 37 °C and 80 rpm to reach constant temperature, then one piece DES was immersed in the solution for 28 days. The solution was replaced with a fresh solution at 3 h, 24 h, 7 days, and 14 days. HPLC (Agilent series 1100, Santa Clara, CA, USA) was used to test drug content of each replaced solution. The initial drug content of a drug-eluting stent was calculated by coating weight and drug/polymer ratio, and accumulated release rates for the above different times were calculated. Three pieces DES as a group were made for drug release profile measurements.

Accumulated release rate (%) at time x = (total drug contents of all replaced solutions at time x/initial drug content of a drug-eluting stent) × 100%.

### 2.6. Polymer Crystallinity Measurement

#### 2.6.1. Samples Made for Testing

All samples (a: 1.8 g P34HB-1, 100 mL CHCl_3_; b: 1.8 g P34HB-1, 100 mL CHCl_3_/NPA (10:1); c: 1.8 g P34HB-10, 100 mL CHCl_3_; d: 1.8 g P34HB-10, 100 mL CHCl_3_/NPA (10:1)) were added to a beaker, respectively, and then stirred for several minutes until clear. After the solvents evaporated, the P34HB films stayed at the bottom of the beakers. Finally, each piece of P34HB film was cut into three pieces with a thickness of about 1 mm.

#### 2.6.2. Crystallinity Measurement

An X-ray diffractometer (Rigaku Ultima IV) was used to test P34HB crystallinity.

### 2.7. Polymer Viscosity Measurement

#### 2.7.1. Samples Made for Testing

All samples (a: 1.8 g P34HB-1, 0.18 g RAPA, 100 mL CHCl_3_; b: 1.8 g P34HB-1, 0.18 g RAPA, 100 mL CHCl_3_/NPA (10:1); c: 1.8 g P34HB-10, 0.18 g RAPA, 100 mL CHCl_3_; d: 1.8 g P34HB-10, 0.18 g RAPA, 100 mL CHCl_3_/NPA (10:1)) were added to a beaker, respectively, and then stirred for several minutes until clear. Each sample was divided into three parts for testing.

#### 2.7.2. Viscosity Measurement

A BROOKFIELD Digital Viscometer (LVDV-C type) was used to test the viscosity of the P34HB solutions.

### 2.8. Cytotoxicity Test

#### 2.8.1. Samples Made for Testing

P34HB (0.5 g) and 10 mL CHCl_3_ were added to a beaker then stirred for several minutes until clear. After the solvent evaporated, the P34HB films stayed at the bottom of the beaker. Finally, the P34HB films were sterilized by ethylene oxide (EO) and cut into three pieces of 39.26 cm^2^ each with a thickness of less than 0.5 mm.

#### 2.8.2. Test Method

One thousand L929 cells in a 100 μL cell culture medium was seeded into each well of a 96-well cell culture plate at 37 °C and 5% CO_2_ for 24 h. Later, the cell culture media were discarded. A blank control group (100 μL of the same batch of cell culture medium), a negative control group (100 μL of the same batch of cell culture medium after the high density polyethylene was extracted), a positive control group (100 μL of the same batch of cell culture medium after 5% DMSO solution was extracted) and the sample group (6 cm^2^/mL, 100 μL of the same batch of cell culture medium after the sample was extracted) were used in contact with the L929 cells for 72 h, then 20 μL (5 g/L) MTT was added for 4 h to each. Later the solvents were discarded and 150 μL DMSO was added and the absorbance was measured at a wavelength of 500 nm using a microplate reader. Relative growth rates were calculated as RGA = (A/A_0_) × 100%, where A: is the absorbance of sample, negative control group, or positive control group, and A_0_: is the absorbance of the blank control group.

### 2.9. Hemolysis Test

#### 2.9.1. Samples Made for Testing

P34HB films as in Section 2.8.1 were sterilized by EO and then cut into three pieces 6 cm^2^ each with a thickness of less than 0.5 mm.

#### 2.9.2. Test Method

At room temperature, the samples were immersed in normal saline (6 cm^2^/mL), then immersed in a 37 °C water bath for 30 min with the negative control group (normal saline) and positive control group (distilled water). Subsequently, fresh anticoagulant rabbit blood was added into the three groups in a proportion of 20 μL/1 mL and the samples were immersed in a 37 °C water bath for 60 min. Later, they were transferred into new centrifugal tubes and the supernatants taken out after centrifugation for 5 min. Finally, supernatant absorbance was measured at a wavelength of 515 nm using a microplate reader and the hemolysis rate was calculated. Hemolysis rate = (OD of sample − OD of negative group)/(OD of positive group − OD of negative group).

## 3. Results and Discussion

### 3.1. P34HB Solutions

#### Observation of Solubility of P34HB

To spray P34HB coatings on stents, a clear and transparent P34HB solution must be prepared, which is determined by the solubility of P34HB in a solvent. Generally, the coatings surfaces are smooth after ultrasonic spraying if the polymer is well dissolved in a solvent. Otherwise, due to the poor solubility of the polymer in solution, the produced coatings surfaces become rough and uneven, and the contact surfaces between the coating and blood become larger. In this case, it is easier to absorb substances in the blood and cause damage to red blood cells [14,15], resulting in poor blood compatibility. Because of coating unevenness, the binding force of the coating to the stent is reduced, increasing the risk of particles falling from the coating into the blood after implantation and causing serious negative effects on the body.

To achieve a clear and transparent polymer solution, the solvent is most important. According to the compatibility principle of solubility parameters, solubility in the solvent is greater if the solubility parameter (δ value) of P34HB polymer is near to that of the solvent [16]. For the dissolution of polar polymers, the solubility parameters not only consider the dispersion force between the two molecules, but also consider the action of polar groups and hydrogen bonds, as expressed in Equation (1) [16]. There are many methods to calculate solubility parameters. The most commonly used basic method is the group contribution method [17,18].

δ^2^ = δ_D_^2^ + δ_P_^2^ + δ_H_^2^ (1)

δ_D_ = (ρ × ∑G_Di_)/M, δ_P_ = (ρ × (∑G_Pi_)^1/2^)/M, δ_H_ = (ρ × ∑E_Hi_)^1/2^/M.

M: Sum of molecular weights of all repeating units for P34HB.

ρ_P34HB-1_ = 1.240 g/cm^3^, ρ_P34HB-10_ = 1.224 g/cm^3^, M = 172 g/mol.

Table 1 shows G/E values of each repeating unit for P34HB. Thus, δ values of P34HB-1 and P34HB-10 are calculated as 17.03 J^1/2^/cm^3/2^ and 16.81 J^1/2^/cm^3/2^, respectively, which are close to that of CHCl_3_ (19.0 J^1/2^/cm^3/2^).

**Figure 2:**
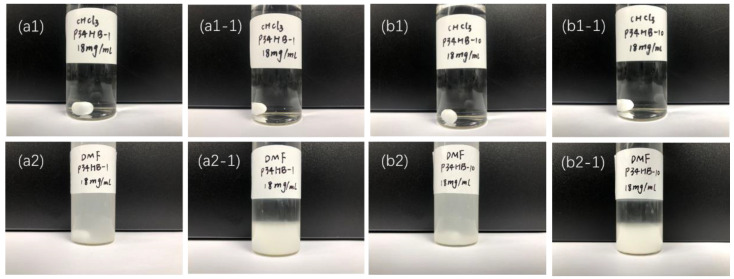
Solubility of P34HB-1 and P34HB-10 in CHCl_3_ and DMF. (**a1**,**b1**,**a2**,**b2**) The state of the P34HB solution just after preparation. (**a1-1**,**b1-1**,**a2-1**,**b2-1**) The state in which the P34HB solution was prepared and placed for 48 h.

As a result, clear and transparent polymer solutions could be obtained within several minutes after P34HB dissolved into CHCl_3_ (Figure 2(a1,b1)). However, even after 4 h stirring, turbid solutions still existed when P34HB was added to DMF (δ = 24.8 J^1/2^/cm^3/2^) as shown in Figure 2(a2,b2), which indicates the solubility parameter principle. Therefore, CHCl_3_ is more suitable for preparing P34HB solutions, and facilitates smooth and high adhesive coatings on stents after ultrasonic spraying.

The stability of a polymer solution is very important for the drug release performance of the stent coatings. If the polymer solution is not stable, there will be visible delamination or filaments after a period of time, leading to large fluctuations in drug content and density of the stent coatings processed by different periods, resulting in large fluctuations in drug release of the coatings.

Figure 2(a1-1,b1-1) shows that both P34HB-1 and P34HB-10 solutions in CHCl_3_ remained stable and did not become turbid after being placed statically for 48 h, which is conducive to batch preparation of uniform drug coatings. However, both P34HB-1 and P34HB-10 solutions in DMF (Figure 2(a2-1,b2-1)) had visible delamination after being placed statically for 48 h, and were not suitable for batch production of DES with stable coating weights. Unstable coating weights in a batch leads to large deviations of drug release rates from each stent and affects the treatment effect.

### 3.2. Morphologies of Coating Surfaces

**Figure 3:**
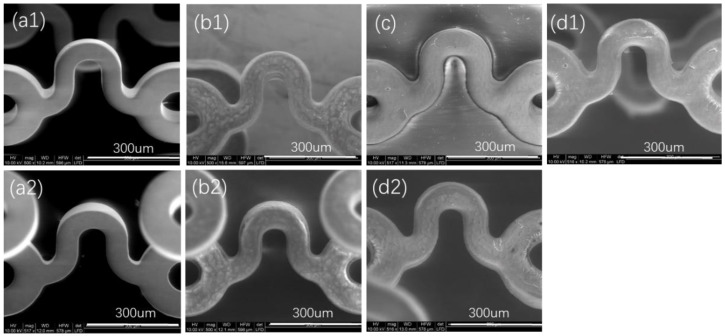
Morphologies of coating surfaces made by P34HB-1. (**a1**) Outer surface of a bare stent; (**b1**) outer surface of a drug stent; (**c**) outer surface of a drug stent at squeezed state; (**d1**) outer surface of a drug stent after expansion; (**a2**) inner surface of a bare stent; (**b2**) inner surface of a drug stent; (**d2**) inner surface of a drug stent after expansion.

**Figure 4:**
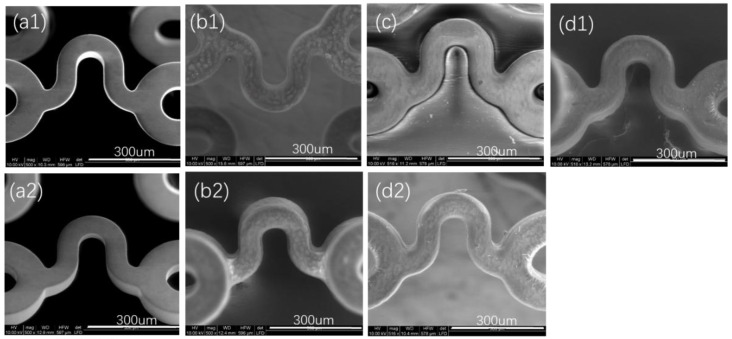
Morphologies of coating surfaces made by P34HB-10. (**a1**) Outer surface of a bare stent; (**b1**) outer surface of a drug stent; (**c**) outer surface of a drug stent at squeezed state; (**d1**) outer surface of a drug stent after expansion; (**a2**) inner surface of a bare stent; (**b2**) inner surface of a drug stent; (**d2**) inner surface of a drug stent after expansion.

Figure 3 shows that both the outer surface and inner surface of a bare stent were smooth, and the coating made by P34HB-1 on the bare stent was also smooth after the stent was sprayed. After the drug stent was squeezed and then expanded, the surface of the coating on the stent remained intact and smooth. Figure 4 shows similar results for the coating made by P34HB-10 on another bare stent. Here, smooth surfaces of the coatings were attributed to P34HB’s excellent solubility in CHCl_3_, and the intact coatings after squeezing and expansion suggested that P34HB had excellent binding to bare stents. The coatings made by P34HB had smooth surfaces that would result in excellent blood compatibility for the stents [15]. Coatings on the stents were so complete, no matter the squeezed state or the expansion state, that they would meet clinical demands of stent binding forces. The extrusion resistance properties were so effective that they would not release harmful particles after implantation in blood vessels [15].

### 3.3. Drug Release Profiles of the Coatings

For a drug-eluting stent, the drug is loaded within the polymer coating of the metal stent. The drug is eluted and released into the blood vessel, creating a high drug concentration around the coronary tissue where the stent is implanted to prevent post-implantation restenosis. In this study, the drug release profiles from the P34HB drug stents and Firebird 2^®^ drug stents were investigated.

**Figure 5:**
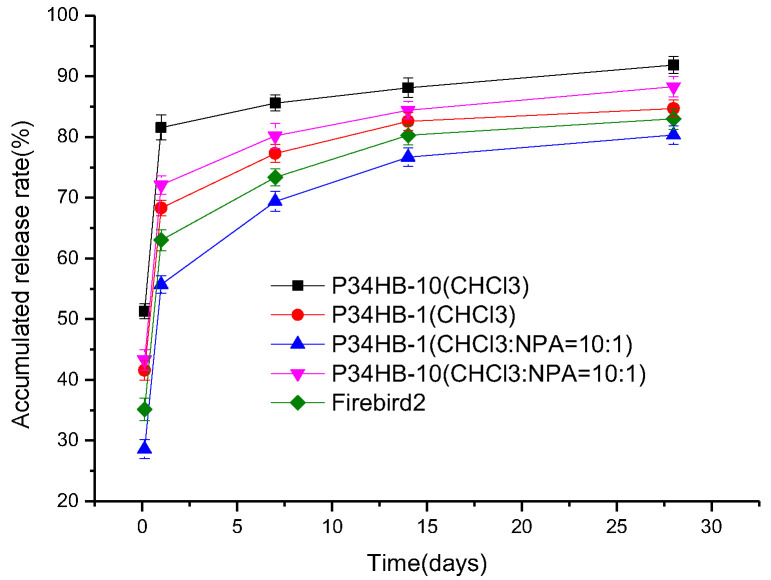
Drug release profiles for P34HB coating stents and Firebird2^®^ stents.

**Figure 6:**
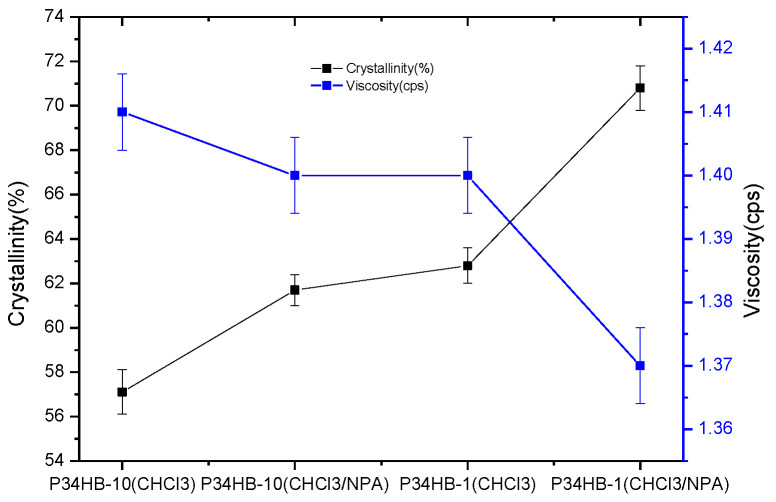
Crystallinity and viscosity for P34HB-1 and P34HB-10.

As shown in Figure 5, both P34HB-1 and Firebird2^®^ stents showed no more than 70% drug release within one day; the latter released drug amount would be enough for effective treatment, while the drug release amount from P34HB-10 stents within one day was more than 80%. The main reason of this could be more crystallinities in P34HB-1 (62.8%) compared to P34HB-10 (57.1%), as shown in Figure 6. P34HB is a thermoplastic crystalline polymer material, and its crystallinity varies with the content of 4HB. A high content of 4HB will not have crystallization characteristics and results in a rubber polymer [10]. More crystallinities meant that more polymer molecular chains are compact and it is more difficult for drug molecules to cross over the polymer molecular chains. Neither P34HB-1 nor Firebird2^®^ stents had sudden drug release phenomena. According to the relevant literature [19], the sudden release phenomenon refers to a situation in which the total drug release exceeds 70% within 24 h after stent implantation, resulting in the effective drug concentration being too low in the later release period, and unable to achieve the therapeutic effect.

P34HB-1 stents had faster drug release rates compared to Firebird2^®^ stents within the whole release period from 0 to 28 days, suggesting that Firebird2^®^ stents had better drug release control for a longer release period. This may be attributed to the relatively hard molecular structure of the Firebird2^®^ polymer. The polymer for Firebird2^®^ is poly (styrene-b-isobutylene-b-styrene) (SIBS, a triblock copolymer). The PS molecular chains in SIBS are alternately connected with side phenyls. Due to the large volume of the side phenyls, they have large steric effects, which makes the PS molecular chains rigid. Such a rigid molecular structure of PS in SIBS might effectively reduce the speed of RAPA crossing SIBS, while there are no such phenomena in P34HB.

Achieving a slower drug release rate by increasing the polymer’s crystallinity by changing its film-forming process, without changing molecular structure, may be an important approach. The relevant literature [20] shows that two solvents with similar polarities are easier to induce PLA to produce higher crystallinity than a single solvent. Mixed solvents with similar polarities can play an important role in promoting the movement of polymer molecular segments, which is conducive to the orderly accumulation of these segments and the growth of crystallization chains, expanding the area of the crystallization zone and increasing crystallinity. The greater the polarity difference of the mixed solvents, the lower the crystallinity. A two-solvent model was introduced in this experiment. It was assumed that there were two kinds of solvents with similar polarities mixed in the P34HB drug solution. The polarities of CHCl_3_ and NPA were 4.4 and 4.0, respectively.

As shown in Figure 5, P34HB-1 stents using mixed solvents (CHCl_3_:NPA = 10:1) had slower drug release rates compared to P34HB-1 stents using CHCl_3_ within the whole release period from 0 to 28 days, as with P34HB-10 stents. The main reason may be the higher crystallinities of both P34HB-1 and P34HB-10 in mixed solvents than in pure CHCl_3_. Figure 6 shows that P34HB-1 and P34HB-10 had higher crystallinities of 70.8% and 61.7%, respectively, in mixed solvents than in pure CHCl_3_. This suggests that there were more orderly accumulations of polymer molecular segments in both P34HB-1 and P34HB-10, and thus their drug coatings became more compact because of use of mixed solvents rather than pure CHCl_3_. Figure 6 also shows that both P34HB-1 and P34HB-10 had lower viscosities in mixed solvents than in pure CHCl_3_, and this lower viscosity promoted the increase of crystallinity. Thus, the two solvent model was successfully introduced and established.

There are many mathematical models applicable to ordinary sustained-release or controlled-release preparations. Some are based on the derivation of drug release principles, some are empirical formulas based on long-term test data, and others are from the perspective of statistics. The literature [21] lists the following main drug release mathematical models that may be applicable to DES: first order model, Higuchi model, Weibull model, Ritger-Peppas model, diffusion-relaxation model and comprehensive model. Compared with other models, the comprehensive model fitted in this experiment for P34HB-1 stents, as in Equation (2), had the best fit (adj.R^2^ = 0.986), and the model was most suitable for the study of drug release kinetics. The fundamental reason is that the model is a drug release model combined with a diffusion-relaxation model and a corrosion (Ritger-Peppas) model. Compared with other models, it considers more factors to fit the drug release data more accurately. The coefficient of x^1/2^ is related to pure Fick diffusion, the coefficient x is related to the phenomena of corrosion and relaxation, and the coefficients x^2^ and x^3^ are related to the phenomenon of corrosion. The coefficient values of both x^1/2^ (85.24647) and x (−29.42659) were so much larger than other coefficients, showing that among various factors, the diffusion factor was dominant and the relaxation (swelling) factor was secondary, which explains the first sudden increase, then the slow increase of P34HB-1’s drug release profile in Figure 5, while the coefficient values x^2^ and x^3^ were negligible, indicating that the corrosion factor had little influence on the drug release behaviors of the stents.

y = 85.24647x^1/2^ − 29.42659x + 1.15482x^2^ − 0.0206x^3^ (2)

P34HB-1 stents using mixed solvents (CHCl_3_:NPA = 10:1) had slower drug release rates compared to Firebird2^®^ stents within the whole release period from 0 to 28 days, suggesting that P34HB-1 stents using mixed solvents had better drug release control for a longer release period. Therefore, P34HB-1 is a latent suitable polymer for drug release coatings.

### 3.4. Biocompatibility

The coating material on a stent should have excellent biocompatibility, especially with respect to cytotoxicity and hemolysis properties, after implantation in a blood vessel.

Cytotoxicity between a coating material and cells refers to the ability of the material to allow cells to adhere and grow on its surface. It is generally believed that cytotoxicity is due to a protein-mediated adhesion mechanism between cells and the material; the difference in cell adhesion affects cell growth and differentiation [22].

Direct contact with blood after implantation of a cardiovascular stent affects the components of blood, such as platelets, red blood cells, white blood cells and plasma proteins. Interaction can lead to thrombosis, hemolysis, activation of the complement system, and changes in visible components in blood. Blood incompatibility of a material seriously endangers human life. Therefore, blood compatibility of a coating material should be in a range compatible with the function and properties of blood or blood components without causing hemolysis [22].

P34HB-1 and P34HB-10 were used to test biocompatibility with respect to cytotoxicity and hemolysis.

**Figure 7:**
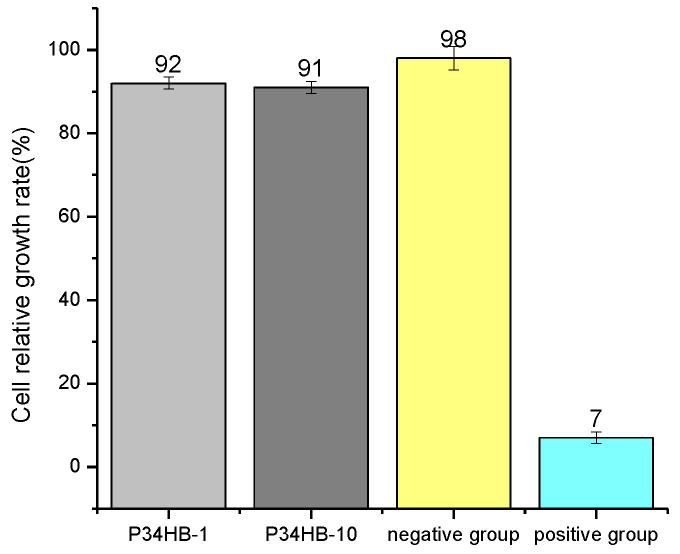
Cell relative growth rates for P34HB-1 and P34HB-10.

Figure 7 shows that cell relative growth rates for P34HB-1 and P34HB-10 were 92% and 91%, respectively, which were close to negative group sample but much higher than positive group sample. These high values of cell relative growth rates indicate that both P34HB-1 and P34HB-10 could allow cells to easily adhere and grow on the surface of the negative group sample, while in the positive group sample there was greater difficulty because of the low value of cell relative growth rate. This indicates that the cellular poison levels of both P34HB-1 and P34HB-10 were reached first, because their relative cell growth rates were between 80~99%.

**Figure 8:**
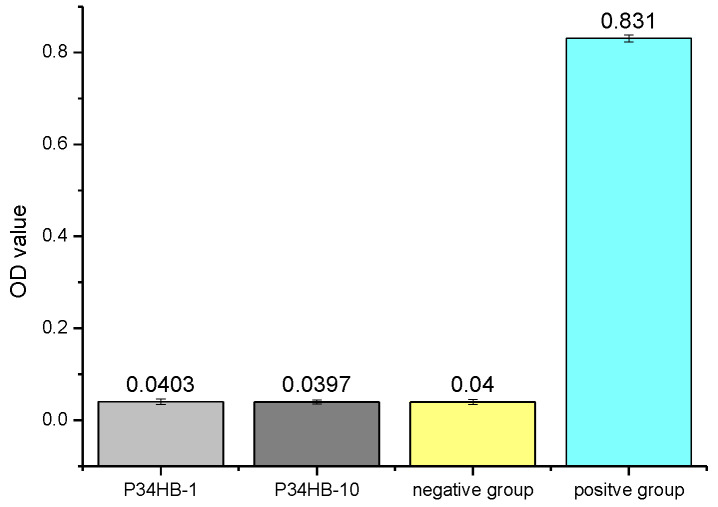
OD values of hemolysis rates for P34HB-1 and P34HB-10.

Hemolysis rates of both P34HB-1 and P34HB-10 were much lower like the negative group sample than that of the positive group sample, as shown in Figure 8. This indicates that both P34HB-1 and P34HB-10 would not affect the function and properties of blood or blood components and had good blood compatibility.

Hence, both P34HB-1 and P34HB-10 are biocompatible materials based on the results of their cytotoxicity and hemolysis tests and could be latent implantable polymers for drug eluting stents.

## 4. Conclusions

In summary, both P34HB-1 and P34HB-10 were proposed as coating polymers for drug eluting stents. CHCl_3_ could dissolve P34HB polymers to form stable drug solutions, which was conducive to the batch preparation of uniform drug coatings. Drug coatings made by both P34HB-1 and P34HB-10 on stents were almost intact before and after dilation by balloon, due to the excellent adhesion and extrusion resistance properties of P34HB polymers. Furthermore, P34HB-1 and P34HB-10 polymers are biocompatible materials, but P34HB-1 polymer has better drug release control than P34HB-10, and shows great promise as a latent coating polymer for coronary stents.

## Figures and Tables

**Table 1:** G/E values of each repeating unit for P34HB.

Repeating Unit	Number	G_Di_ (J^1/2^ cm^3/2^ mol^−1^)	G_Pi_ (J^1/2^ cm^3/2^ mol^−1^)	E_Hi_ (J^1/2^ cm^3/2^ mol^−1^)
-COO-	2	390	490	7000
-CH_2_-	4	270	0	0
>CH-	1	80	0	0
-CH_3_	1	420	0	0

## Data Availability

Data available on request due to restrictions e.g., privacy or ethical. The data presented in this study are available on request from the corresponding author. The data are not publicly available due to later possible commercial uses.
